# A Snapshot of the Global Trade of South African Native Vertebrate Species Not Listed on CITES

**DOI:** 10.3390/ani14192782

**Published:** 2024-09-26

**Authors:** Ndivhuwo Shivambu, Tinyiko Cavin Shivambu, Takalani Nelufule, Moleseng Claude Moshobane, Nimmi Seoraj-Pillai, Tshifhiwa Constance Nangammbi

**Affiliations:** 1 Department of Nature Conservation, Faculty of Science, Tshwane University of Technology, Private Bag X680, Pretoria 0001, South Africa; shivambutc@tut.ac.za (T.C.S.); nelufulet@tut.ac.za (T.N.); seorajpillayn@tut.ac.za (N.S.-P.); nangammbitc@tut.ac.za (T.C.N.); 2 South African National Biodiversity Institute, Pretoria National Botanical Garden, 2 Cussonia Avenue, Brummeria, Silverton 0184, South Africa; m.moshobane@sanbi.org.za

**Keywords:** pet trade, wildlife products, illegal trade, international cooperation, overexploitation, IUCN status

## Abstract

**Simple Summary:**

Understanding the wildlife trade is crucial for effective conservation and policy development, particularly in South Africa, where native species face exploitation as exotic pets and wildlife products. The trade, influenced by socioeconomic factors, presents challenges in balancing conservation with local livelihoods. Our study highlights the gaps in current regulations, especially for non-CITES-listed species, and emphasizes the need for comprehensive data collection, stricter legal frameworks, and community involvement to protect vulnerable species while supporting sustainable practices.

**Abstract:**

The Convention on International Trade in Endangered Species of Wild Fauna and Flora (CITES) aims to prevent the overexploitation of species by controlling their trade. However, there is currently no international regulatory framework to protect the trade of non-CITES species. We examined the LEMIS database, online trade, and scientific literature with the aim of identifying and compiling a list of South African native species traded as pets and wildlife products. We found that there are 223 non-CITES species traded as wildlife products and 95 species traded as pets. Mammals and birds were the most traded taxa for wildlife products, while reptiles and amphibians were mostly traded as pets. At the least, species traded as wildlife products and pets are currently not facing extinction, as most are categorized as Least Concern. However, some endemic species have an unknown population size, with *Sclerophrys pantherina* and *Neamblysomus gunningi* being Endangered. The international pet trade involves 10 countries, with the USA, the Czech Republic, and the UK being the largest importers. The trade of species as wildlife products involves 20 countries, with the USA being the major importer. This study emphasizes the necessity of strict regulations and international cooperation to control the wildlife trade effectively.

## 1. Introduction

Wildlife trade involves the sale of live and dead animals as pets, medicine, meat, and ornaments or trophies [1,2,3]. The global wildlife trade is a multibillion-dollar industry which involves the exchange of millions of animals between countries annually [2,4,5,6]. There has been a significant increase in research focusing on the global wildlife trade over the past decade, particularly the trade of live animals as exotic pets [7,8,9]. This increase may be explained by the international recognition of the threats posed by unsustainable trade practices, which has resulted in criminal activities (e.g., illegal wildlife trade) and, subsequently, overexploitation of species in their native ranges [10,11,12,13]. Overexploitation may reduce species diversity and abundance [2,14,15]. For example, the International Union for Conservation of Nature (IUCN) has reclassified two African pangolin species, the giant ground pangolin (*Smutsia gigantea*) and white-bellied pangolin (*Phataginus tricuspis*) from Vulnerable to Endangered as a result of the wildlife trade [16,17].

The wildlife trade has also been identified as a significant contributor to the spread of invasive alien species and zoonotic diseases [18,19,20]. For example, species such as the giant African snail (*Achatina fulica*) and Mozambique tilapia (*Oreochromis mossambicus*) are listed as one of the top 100 world’s worst invasive alien species [21]. On the other hand, the outbreak of monkeypox in the United States of America (USA) in 2003 was associated with the domestic trade of species such as Gambian giant rats (*Cricetomys gambianus*), dormice (*Glis* spp.), and striped mice (*Rhabdomys* spp.) that are indigenous to Africa [22,23,24]. These cases highlight the vital need for stricter regulations and international cooperation to control the wildlife trade. These regulations should include conducting risk assessments to determine the potential invasiveness of the species and screening for diseases before the exportation or importation of species.

The African continent serves as a major source of both legal and illegal wildlife trade exports, and, as a result, it plays a major role in the global wildlife trade [25,26]. The wildlife trade is facilitated by the Convention on International Trade in Endangered Species of Wild Fauna and Flora (CITES), which is an international agreement between governments, designed to ensure that global trade in wild animals and plants does not jeopardize their survival [https://cites.org/eng/disc/text.php, accessed on 9 September 2024]. CITES has three appendices: Appendix I lists species that are at risk of extinction and prohibits international trade in these species, except in rare and exceptional cases [https://cites.org/eng/app/index.php, accessed on 9 September 2024]. Appendix II lists species not necessarily threatened with extinction but for which trade must be controlled to avoid utilization incompatible with their survival [https://cites.org/eng/app/index.php, accessed on 9 September 2024]. Appendix III includes species protected in at least one country which has asked other CITES parties for assistance in controlling trade [https://cites.org/eng/app/index.php, accessed on 9 September 2024]. The inclusion of species in these appendices is determined by proposals submitted by member countries, which are then reviewed and voted on during the Conference of the Parties (CoP) [27]. While CITES provides a framework, it sets minimum requirements, leaving it up to individual signatories to implement stricter domestic measures if they choose [27]. Africa, home to a significant number of CITES-listed species, plays a vital role in the enforcement and promotion of CITES regulations [28].

A study by Smith et al. [29] found that the most common origins of live herpetofauna and avifauna species were in Africa, Asia, and South America. Even though most African countries are signatories to CITES, some still struggle with persistent challenges associated with extensive poaching and illegal wildlife trade [25]. For example, South Africa is amongst the top five countries with the highest number of illegal wildlife trade reports, with 75 species involved [15]. It is important to note that some of the native South African species traded in the international market are not listed on CITES [20,30]. Consequently, this highlights the complexity and challenges in regulating the trade of such species. These challenges are exacerbated by the lack of international legal frameworks for non-CITES species. As a result, this may create loopholes for illegal trade activities, particularly in social media and online [9,31,32].

Non-CITES species may not receive the same level of regulatory inspection as those listed under CITES, and, as a result, this may complicate efforts to monitor and manage their trade, given that most of them are not protected in their countries of origin [31,33]. Furthermore, there is a lack of standardized regulations across countries, as some nations fail to report their wildlife trade data, leading to inevitable variations in reporting standards across parties [4,5]. There are also variations in trade regulations within provinces or states. In South Africa, each province has its own legislation regarding which wildlife species to trade ([34], https://cer.org.za/virtual-library/legislation, accessed on 6 September 2024). For example, keeping or collecting indigenous reptile species is prohibited in certain provinces of South Africa, while it is allowed in others ([35], https://cer.org.za/virtual-library/legislation, accessed on 7 September 2024). The National Environmental Management: Biodiversity Act, 2004 (Act No. 10 of 2004) specifies which species are protected and threatened. In addition, those species are protected from hunting, catching, capturing, and also killing.

South Africa boasts a rich biodiversity and is home to a diverse range of flora and fauna that has captured the fascination of people worldwide [36,37]. However, the trade of native animals as pets and sources of wildlife products raises complex questions about sustainability and animal welfare [8,38,39]. Understanding the dynamics of wildlife trade is essential in limiting illegal or unsustainable practices [40], particularly for species not listed in CITES. In this study, we intentionally focused exclusively on species not listed under CITES. Our rationale for this approach is that species listed on the CITES database are well documented and accessible, while there is a gap in the literature concerning non-CITES-listed species in South Africa. Our aim was, therefore, to determine which South African non-CITES-listed native vertebrate species are sold as pets and/or wildlife products. Additionally, we aimed to identify which countries import South African species not listed on CITES, and for which purpose (pets or wildlife products). Lastly, we evaluated their IUCN and protected status in South Africa. This study will contribute to informed wildlife trade regulation, promoting biodiversity conservation and protection of endemic and threatened species in South Africa. We believe that investigating non-CITES species will provide new insights and complement existing data on wildlife trade in South Africa.

## 2. Materials and Methods

### 2.1. Data Collection

#### 2.1.1. US Fish and Wildlife Service (USFWS) Law Enforcement Management Information System (LEMIS)

We compiled import and export data from the LEMIS database spanning 2000 to 2014, focusing on specific criteria. Our data selection involved filtering for the following: (1) country of origin (South Africa) and import and export countries; (2) taxa, including amphibians, birds, mammals, and reptiles; (3) non-CITES species; and (4) species traded for all purposes except scientific research. Species traded for the pet trade were identified based on specific labels denoting commercial purposes (T), breeding in captivity (B), and personal ownership (P). However, only live specimens were considered for pet trade purposes (see inclusion criteria in Table S1). For wildlife products, species with trade descriptions related to animal products were included (Table S1). We excluded fish data from the LEMIS database because there was only one non-CITES South African native species recorded. As a result, this would have made it impossible to analyse and compare. Species labelled T, B, or P were not automatically assumed to be traded for pet trade purposes. We verified if the species were pets on the International Union for Conservation of Nature (IUCN) (https://www.iucnredlist.org/, accessed on 6 May 2024) list. We checked for the use and trade of the species; if these were indicated as pets or display animals, then we concluded that the species were traded as pets.

#### 2.1.2. Online Survey

We surveyed the list of websites from Choquette et al. [41], and we also searched for other websites (Table S2) using the Google search engine to compile a list of species sold as pets. Google searches were conducted from September 2023 to February 2024. We used the following keywords: “exotic mammal pets for sale”, “exotic amphibian pets for sale”, “exotic reptile pets for sale”, and “exotic pet birds for sale”. We also used Google Maps to search for online pet shops in each country; this search resulted in a list of exotic pet shops in different countries. We navigated to each pet shop and searched for the list of amphibians, birds, mammals, and reptiles offered for sale. For pet shops using non-English languages, lists were automatically translated into English, e.g., for the Czech Republic. After compiling a list from the pet shops, we used the list of pets from the LEMIS database to search for each species sold; for example, we used keywords such as “African bullfrog for sale”, “puff adder for sale”, “African pygmy mouse for sale”, and “hamerkop for sale”. We did this for each amphibian, bird, mammal, and reptile species to ensure that we included all the species. Most websites provided both common and scientific names, making it easier to accurately identify the species being sold. For sites that listed only common names, we relied on accompanying photographs to identify the species and cross-referenced them with biodiversity databases to confirm their scientific names. We also searched for species sold as wildlife products online. We used the following keywords to search for species sold as wildlife products online: “mammal skull for sale”, “reptile skin for sale”, “bird wings for sale”, “amphibian bones for sale”, and “animal teeth for sale”. However, we did not use the online data because there were very few websites selling wildlife products, and those that did only listed generic names such as “mammal skull for sale” or “reptile skin for sale” without specifying the species.

#### 2.1.3. Literature Review

In combination with online data collection and the LEMIS database, we conducted a literature review to identify species traded as pets and wildlife products (Table S3). We conducted our searches using the following academic databases: (1) Web of Science (https://www.webofscience.com/wos/, accessed on 7 May 2024); (2) Scopus (https://www.scopus.com, accessed on 7 May 2024); (3) PubMed (https://pubmed.ncbi.nlm.nih.gov/advanced, accessed on 7 April 2024); and (4) Google Scholar (https://scholar.google.com/, accessed on 7 March 2024). A Boolean search strategy was employed by combining search terms or phrases with AND/OR in the Web of Science, PubMed, and Scopus query boxes. The search keywords or phrases included: “pet”, “reptile or bird or mammal or amphibian”, “pet shop”, “online trade”, “exotic pets”, and “international/global trade”.

For Google Scholar, we searched the articles using the terms “exotic pet amphibian/bird/mammal/reptile trade”, “the trade of amphibians/birds/mammals/reptiles as exotic pets”, “the trade of pet amphibian/bird/mammal/reptile on online or on the internet”, and “pet amphibian/bird/mammal/reptile for sale”. For additional literature review articles, we searched for relevant articles on SciSpace (https://typeset.io/, accessed on 15 April 2024) using similar search keywords to those used on Google Scholar. SciSpace is an academic search engine that uses indexing of scholarly literature from various disciplines for scientific research. The platform functions similarly to Google Scholar but differs in that it offers enhanced filtering capabilities for search results. While both platforms index scholarly literature, SciSpace allows users to filter search results based on specific criteria, such as publication type, date, methods used, abstracts, and summaries. For our study, we filtered it to include insights, where it lists the species sold. This feature enables researchers to refine their searches more effectively and access relevant publications more efficiently than with Google Scholar alone. Papers were exported as RIS, BIB, and NBIB files, which are readable in R statistical software, version 4.1.3 [42]. We followed the screening procedure outlined by Shivambu et al. [24].

#### 2.1.4. Inclusion Criteria

After compiling a list of species sold from the three sources, we searched for their native ranges on the IUCN website database and filtered the species origin to only include those from South Africa. Some online websites indicated the origin of the species sold. For websites where the origin was not mentioned, we checked if relevant studies had been conducted to determine the origin. If a study mentioned the country of origin as South Africa, we assumed that the species sold online were South African, based on the literature. We excluded CITES-listed species, as the data on those species is wildly available, and we wanted to access the gap in the trade of non-CITES-listed species.

### 2.2. Species Identification and Verification

Species identification was verified by cross-referencing with reputable biodiversity databases such as AmphibiaWeb (https://www.calacademy.org/explore-science/amphibiaweb, accessed on 13 May 2024), International Ornithological Congress (IOC) World Bird List [43], Mammal Diversity Database (https://www.mammaldiversity.org/, accessed on the 13 May 2024), and the Reptile Database (http://www.reptile-database.org/, accessed on 13 May 2024). The conservation status of the identified species was determined using the IUCN Red List, and the CITES website was visited to verify whether the species was listed or not.

### 2.3. Data Analysis

All data were analysed in R statistical software, version 4.1.3 [42]. We focused specifically on species and genus as the primary taxonomic unit. Subspecies was not included to maintain consistency and precision in our dataset. We evaluated whether there was a significant difference between the two trade mechanisms, namely wildlife products and pet trade using the Pearson’s chi-square test (χ^2^). Graphical representations of the data were generated using the R software [42]. We generated import and export maps using ArcGIS desktop, version 10.4.3 [44].

## 3. Results

### 3.1. Dataset

We recorded a total of 79 species traded as wildlife products on the LEMIS database, of which 24 were birds, 49 mammals, and 6 reptiles. None of the amphibian species traded as wildlife products were found on the LEMIS database. We found at least 6 species of amphibians traded as pets on the LEMIS database. None of the birds listed on LEMIS were traded as pets, while 5 mammal and 9 reptile species were recorded. Our online survey recorded a total of 8 amphibians, 6 mammals, and 22 reptile pets. However, we did not record any bird species sold as pets on the online survey. We found a total of five scientific papers (publication year from 1998 to 2022) reporting on the trade of native South African species as wildlife products. However, we recorded 180 species, of which 50 were birds, 112 mammals, and 18 reptiles. Amphibians were listed in at least two papers, with one paper listing the species’ scientific name and another labelling amphibians as “unidentified”. For the literature review on the pet trade, we found a total of 22 articles (Publication year from 2005 to 2023) representing 82 species, of which 13 were amphibians, 9 birds, 5 mammals, and 55 reptiles.

**Figure 1:**
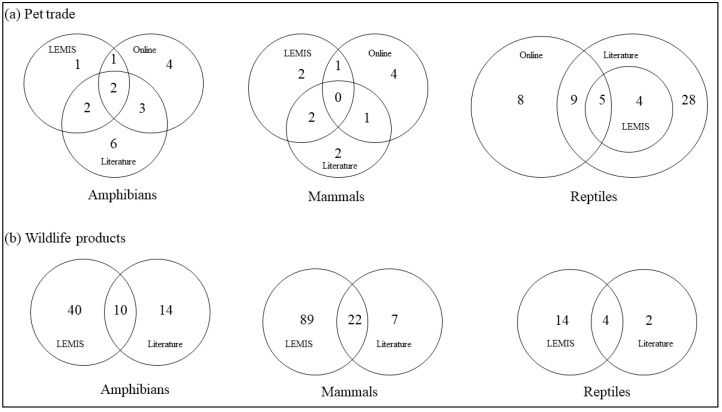
A Venn diagram showing the number of species traded as pets (**a**) and as wildlife products (**b**) shared between sources.

Overall, for the pet trade, the online survey and literature review were the primary sources for most of the species (Figure 1a; Table S4). Two amphibian species were recorded across all sources. The online and LEMIS databases shared one species, while the online and literature review shared three species, and the LEMIS database and literature review shared two species (Figure 1a). For mammals, no species were shared among all three sources; however, online sources had the highest number of unique species (Figure 1a). Regarding reptiles, the literature review identified the most unique species and shared most species with online sources (Figure 1a). For species traded as wildlife products, none of the sources had unique species (Figure 1b). However, the LEMIS database recorded a greater number of species not found in the literature (Figure 1b; Table S5).

### 3.2. Number of South African Non-CITES Traded Species

**Figure 2:**
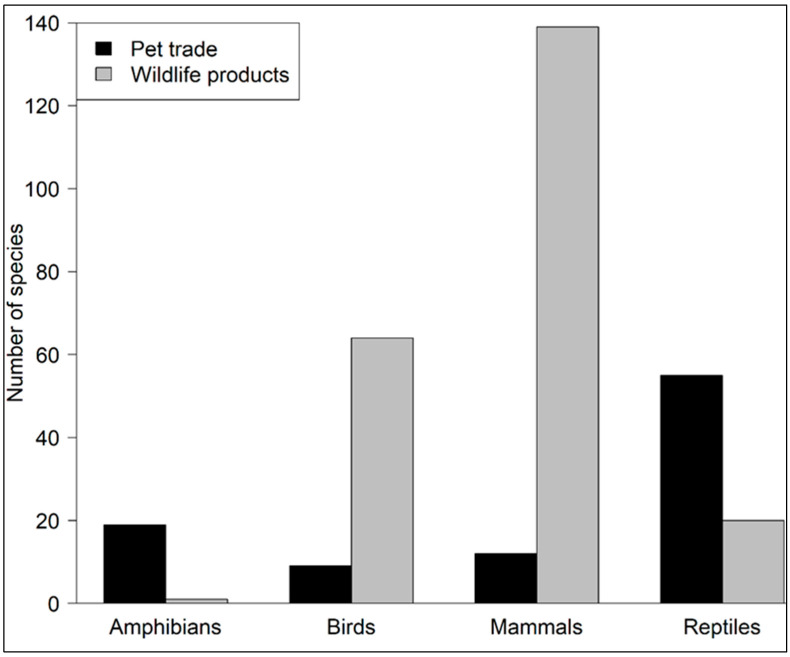
The number of amphibian, bird, mammal, and reptile species traded as wildlife products and exotic pets.

Our study found that the number of South African native taxa listed on CITES (total number of species (n) = 266) was lower than the number of non-CITES taxa (n = 291). None of the South African native amphibian species are currently listed under CITES. However, the number of CITES-listed bird species was 116 compared to 68 non-CITES-listed birds (Figure S1). For mammal species, the number of non-CITES species (n = 143) was two times higher than the number of CITES-listed species (n = 79) (Figure S1). In the case of reptiles, we found that there are 71 CITES-listed species compared to 61 non-CITES-listed species (Figure S1). We found that most of the amphibian, bird, mammal, and reptile species are traded as wildlife products rather than for pet trade purposes (Figure 2). There was a significant difference between the number of species traded as wildlife products and pets (Pearson’s Chi-squared test: χ^2^ = 153.77, df = 3, *p* < 0.05). The number of amphibian, birds, mammal, and reptile species traded as wildlife products (n = 224) was approximately two times higher than the number of species traded for pet trade purposes (n = 95) (Figure 2). We found that 20% of species traded as pets were amphibians (n = 19), 9% (n = 9) birds, 13% (n = 12) mammals, and 58% (n = 55) reptiles. Conversely, for the species traded as wildlife products, amphibians only comprised 0.4% of the trade. However, mammals were the most traded group with 62.1% (139 species in total); this was followed by birds with 28.9% (n = 64) of the species traded, and reptiles with only 8.9% (n = 20). Overall, birds and mammals are mostly traded as wildlife products rather than as pets (Figure 2).

### 3.3. Species Traded

All amphibian species traded as pets belong to 1 order, with Hyperoliidae, and Bufonidae being the dominant families (Table S4). The most traded amphibians were from the least recorded families, including Pyxicephalidae (*Pyxicephalus adspersus*) and Pipidae (*Xenopus laevis*). Only one species was recorded for the trade in wildlife products, *Schismaderma carens*, order Anura and family Bufonidae (Table S5). For pet birds, we recorded 5 species orders and 7 families, while 14 orders and 31 families were recorded for the wildlife products (Tables S4 and S5). We did not find any commonly traded species of birds traded as both pets and wildlife products (Tables S4 and S5). We recorded 2 species orders and 8 families for the mammal pet trade, while for wildlife products, we recorded 8 species orders and 21 families (Tables S4 and S5). Order Rodentia was common, with *Graphiurus murinus* being the most traded species (Table S4).

For the trade in wildlife products, order Chiroptera was overly represented, with 55 species (Table S5); however, family Bovidae was most recorded. The most traded species for wildlife products included *Phacochoerus africanus* and *Aepyceros melampus* (Table S5). Reptiles traded as pets and wildlife products were represented by 2 species orders (Squamata and Testudines), with Gekkonidae and Elapidae being the most common families, respectively (Tables S4 and S5). The most dominant pet species included *Bitis arietans*, *Gerrhosaurus flavigularis*, and *Aspidelaps lubricus* (Table S4). For wildlife products, the most common species included *B*. *arietans*, *Dendroaspis polylepis*, and *Hemachatus haemachatus* (Table S5).

### 3.4. IUCN and Protection Status

We found that 93% (n = 74) of the species traded as pets are categorized as Least Concern, while 4% (3) are Not Evaluated (*Platysaurus janssoni*, *Phlyctimantis maculatus*, and *Pelusios gabonensis*), and only 1% (n = 1) are Endangered (*Sclerophrys pantherina*), Near Threatened (*Breviceps macrops*), or Vulnerable (*B*. *gabonica*) (Table S4). A large proportion of the species traded as wildlife products were Least Concern (83.3%, n = 185), while 3.6% (n = 8) were Near Threatened or Not Evaluated (n = 8) and 4.1% (n = 9) Endangered (Table S5). Only 2.7% of the species were Vulnerable, and at least one species (0.5%) was Critically Endangered. Species that were Data Deficient comprised only 2.3% (n = 5), and all these were mammals. In terms of the population trend, we found that most of the amphibian and reptile pet species have unknown population sizes, when compared to other taxa, while for the species traded as wildlife products, most birds and mammals have unknown population sizes.

We found that a total of 15 species traded as pets were endemic, and most of these species were recorded from reptile and amphibian taxa (Table S4). One of the species, *Sclerophrys pantherina* is Endangered with an unknown population size. For the wildlife products, 17 species were endemic, with mammals and reptiles associated with the trade of these species (Table S5). Two of the endemic species traded as wildlife products are Critically Endangered (*Cryptochloris wintoni*) and Endangered (*Neamblysomus gunningi*), both with an unknown population size. We also found that some of the species are listed on the 2005 national draft lists of threatened and protected species issued by the National Environmental Management: Biodiversity Act, 2004 (Act No. 10 of 2004). These species included two amphibians (*Pyxicephalus adspersus* and *Pyxicephalus edulis*), reptiles (*B. gabonica* and *Dasypeltis medici*), and one mammal species (*Vulpes chama*). However, the published NEM: BA lists of threatened and protected species did not include these species, but included five mammals (*Antidorcas marsupialis*, *Connochaetes gnou*, *Canis mesomelas*, *Sylvicapra grimmia*, and *Vulpes chama*) and one bird species (*Laniarius ferrugineus*).

### 3.5. Countries Involved in the Trade of South African Native Species

**Figure 3:**
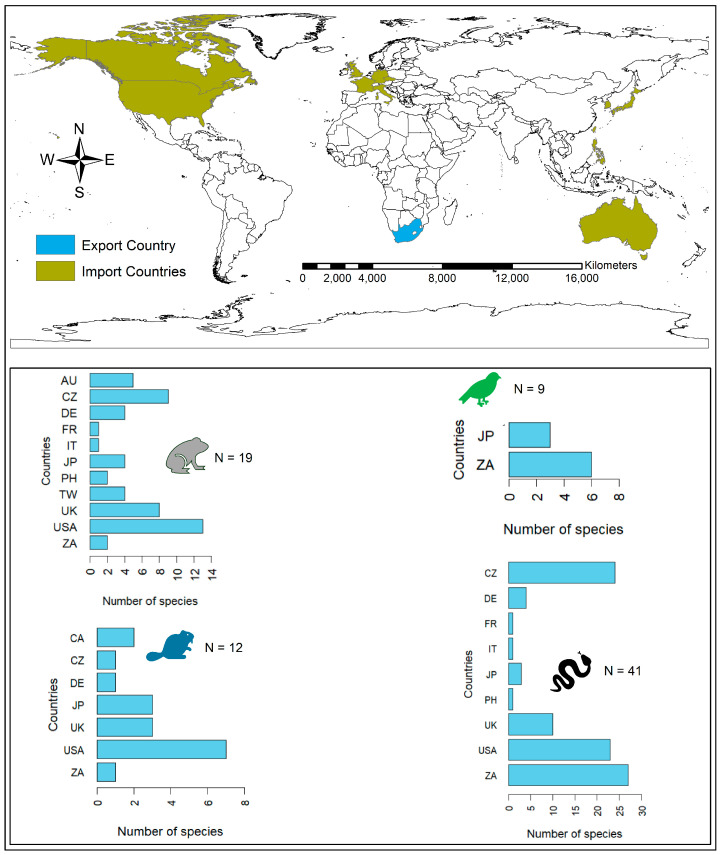
Countries involved in the trade of South African native amphibians, birds, mammals, and reptiles as pets (data obtained from the literature review, online survey, and LEMIS database). Country abbreviations (AU: Australia, CA: Canada, CZ: the Czech Republic, FR: France, DE: Germany, IT: Italy, JP: Japan, PH: Philippines, ZA: South Africa, TW: Taiwan, UK: the United Kingdom, and USA: the United States of America).

**Figure 4:**
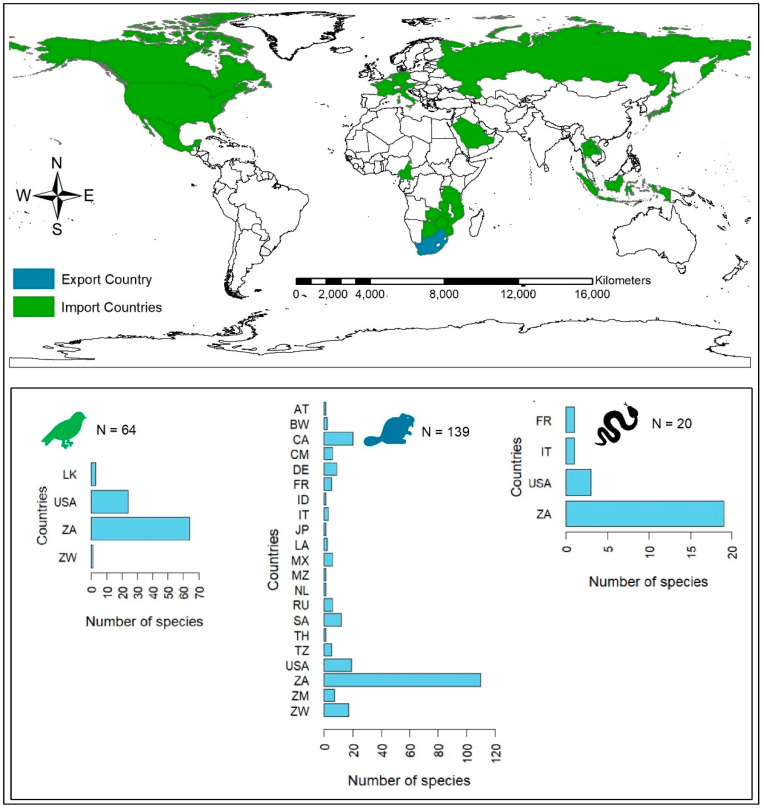
Countries involved in the trade of South African native amphibians, birds, mammals, and reptiles as wildlife products (data obtained from the literature review and LEMIS database). Country abbreviations (AT: Austria, BW: Botswana, CM: Cameroon, CA: Canada, FR: France, DE: Germany, ID: Indonesia, IT: Italy, JP: Japan, LA: Laos, MX: Mexico, MZ: Mozambique, NL: the Netherlands, RU: Russia, SA: Saudi Arabia, ZA: South Africa, LK: Sri Lanka, TZ: Tanzania, USA: the United States of America, ZM: Zambia, and ZW: Zimbabwe).

We found that South African native species are traded locally and internationally as wildlife products and for pet trade purposes (Figure 3 and Figure 4). In the international pet trade, we found that 11 countries are involved, with the USA, the Czech Republic, and the United Kingdom (UK) being the top importers. Amphibians and reptiles were the most imported taxa traded by 11 and 9 countries, respectively. Countries such as the USA, the UK, the Czech Republic, and Australia imported a greater number of amphibians when compared to other countries. However, more species of reptiles were imported, particularly by the Czech Republic and the USA, each with more than ten different species (Figure 3). However, South Africa traded more species of reptiles within the country. The USA also had the highest number of amphibian species traded, when compared to other countries.

For the species traded as wildlife products, we found that a total of 21 countries are involved in the trade of South African species (Figure 4). Most of the species were traded locally in South Africa; however, internationally, the USA was the country importing most species, trading all the taxa except amphibians. We found that this country imports a total of 21 birds, 19 mammals, and 4 reptile species. Overall, mammals were the most traded taxon, traded by more than 10 countries. Locally, more than 100 different species of mammals are traded.

## 4. Discussion

Understanding the scope and scale of wildlife trade is important for effective conservation efforts and policy development, including enforcing laws aimed at protecting native biodiversity [29,45,46]. In the context of South Africa, the trade of native species as wildlife products and exotic pets presents a complex conservation challenge. This challenge is exacerbated by the socioeconomic aspect, as local communities often engage in wildlife trade as a means of making a living [47,48]. Implementing more stringent regulations could have unintended consequences, such as increasing poverty or encouraging environmentally harmful alternatives. Therefore, we suggest that any enhanced monitoring and regulation of non-listed species should be accompanied by support mechanisms, such as providing alternative income sources, community-based conservation programmes, and education initiatives that promote sustainable trade practices. Additionally, collaboration between governments and local communities will be crucial to ensure that conservation efforts do not disproportionately harm vulnerable populations, but rather empower them to participate in wildlife conservation in a sustainable and equitable way.

Our study provides a snapshot of the trade of South African native species not listed in the CITES database. Three sources of data revealed that South African native amphibian, bird, mammal, and reptile species are traded locally and internationally as wildlife products and as pets. The scientific literature reported a greater number of species traded as pets when compared to the LEMIS database. It is evident that the LEMIS database does not record all the species imported by the USA. This could be explained by the fact that the LEMIS database does not record species-specific details, as most are classified under more general codes [49]. On the other hand, online data provide real-time data information on species availability, prices, and market trends [50,51]. However, this platform may not record all the species, particularly those traded on the dark web. Overall, the scientific literature provides valuable insights into the trade of different species. This source is more reliable because real-time surveys are conducted, and the recording of the species is accurate as it is done by experts. This suggests the importance of using multiple sources to gather the full scope of wildlife trade data.

This study specifically documented the trade of species not listed in the CITES database. As a result, this study provides important insights into the extent of the wildlife trade beyond the scope of CITES regulations. Although the information regarding non-CITES-listed species is fragmented, inconsistent, and often incomplete, offering only a limited insight into the actual volumes of trade, this study is important in the context of South African conservation efforts. Our study found that the number of listed taxa was lower than the number of non-CITES-listed taxa. This suggests that several South African species may not benefit from the international regulations and protection under CITES, and, as a result, these species may be more vulnerable to exploitation and unsustainable trade practices. The absence of any South African amphibian species in the CITES database is concerning, given that this taxon faces numerous threats, including habitat destruction, pollution, and emerging infectious diseases [20,52]. Consequently, with the pet and wildlife product trade being another threat, this taxon may be highly susceptible to population decline.

Interestingly, we found a high representation of birds and reptiles on the CITES list when compared to non-CITES-listed species. As a result, this suggests a stronger international regulatory framework for these taxa. However, this is not the same for mammal taxa, as we found that a large number of this taxon are not listed on CITES. This raises concern as mammals also face a range of other challenges, including habitat destruction, poaching, and human-wildlife conflict [53,54]. In addition to the differences in CITES listings, we found that most of the species are traded as wildlife products rather than as pets. However, mammals were the most traded group for wildlife products, followed by birds. These results suggest that mammals and birds are specifically targeted for their parts, likely due to the demand for traditional medicine and trophies [29,55]. Amphibians and reptiles are mostly traded as pets rather than as wildlife products. This observation may reflect the popularity and demand for these two taxa as exotic pets rather than as wildlife products. Amphibians and reptiles were also recorded from most websites, indicating the broader trend in the wildlife trade. South African native birds and mammals are comparatively less popular as pets, which is why most of the websites encountered specialized in reptiles and amphibians. In addition, we only recorded one species of amphibian traded as wildlife products. This could be explained by the potential challenges in identifying amphibian species sold as wildlife products in the wildlife trade markets. For example, one of the studies recorded one amphibian species as “unidentified” [56]. As a result, molecular techniques could be employed to enhance the accurate identification and documentation of amphibian species, including unidentifiable products, in the trade markets. This indicates that there could be challenges in accurately documenting species sold as wildlife products.

Our data showed that species from different orders and families are sold as wildlife products and pets. Interestingly, while certain families, such as Hyperoliidae and Bufonidae for amphibians, are more commonly traded in the pet trade, the most traded species often belong to less recorded families such as Pyxicephalidae (*P*. *adspersus*) and Pipidae (*X*. *laevis*). The preference for these species in trade could be due to their adaptability to captive conditions, which makes them easier to breed and maintain in captivity [57,58]. *Xenopus laevis*, in particular, is known for its widespread use in scientific research, contributing to its high trade volume [59,60]. For birds, we found that there were no commonly traded species as pets or used for wildlife products. The pet bird trade often focuses on species known for their companionship, vibrant plumage, and singing abilities, such as parrots and canaries [12,61]. These traits make them desirable for domestic purposes [61,62]. As a result, South African birds are less commonly traded in the international pet trade because they lack those characteristics. In contrast, birds traded as wildlife products are often valued for their feathers, meat, or cultural significance [63], leading to a different set of species being targeted.

For mammals, order Rodentia was the most common in the pet trade. This may be because most people keep small mammals as pets [64]. Rodents are popular pets due to their small size, ease of care, and breeding capabilities [34,65]. Large and medium-sized mammals, on the other hand, are often killed for wildlife products [66]. Hence, we found that the most common family for wildlife trade products was Bovidae. Gekkonidae and Elapidae for reptiles were the most commonly traded in the pet trade. Dominant pet species such as *B*. *arietans*, *G*. *flavigularis*, and *A*. *lubricus*, are likely common due to their unique appearance. For wildlife products, *B. arietans* is common, potentially due to its use in traditional medicine, venom collection, and leather production [67]. While most species are not on the CITES listing, most are not currently faced with extinction threats. We found that a large percentage of species traded in both pet trade and for wildlife products are Least Concern. Therefore, this indicates that they are not currently considered at high risk of extinction. However, a small percentage of the species traded as wildlife products are either Not Evaluated, Endangered, Near Threatened, and or Vulnerable. As a result, these species require continued monitoring and assessment of their conservation status. In contrast, for the species traded as pets, we found a slightly higher percentage of the species whose statuses include Near Threatened, Endangered, Vulnerable, and Critically Endangered. Of concern, we found 18 and 17 endemic species traded in the pet trade and for wildlife products, respectively. Three of these species have unknown population sizes, with *Sclerophrys pantherina* and *Neamblysomus gunningi* being Endangered and *Cryptochloris wintoni* being Critically Endangered. The presence of these species highlights the need and urgency for their conservation. In addition, species with decreasing or unknown population size may also need to be monitored; for example, we found that most of the species traded as pets have an unknown population size, while others have a decreasing population. Policies should aim to strengthen legal frameworks, enhance enforcement mechanisms, and promote sustainable practices that minimize the negative impacts on wild populations.

South Africa has its own laws that protect the trade of wildlife; however, the law does not protect the majority of the species. For example, only a few species are protected according to the NEM: BA. This may lead to unstainable trade of these species. In addition, each province has its own permitting system regarding the export or trade of native species. For example, a study by van Wilgen et al. [35] found that some of the provinces do not have permit data, while some have loopholes in the law. Some of the provinces only track the species listed on CITES [35]; this suggests that the trade of non-CITES species in other provinces is not traced. Of concern is that species whose conservation status is either Near Threatened or Endangered are not included on the NEM: BA lists of threatened and protected species. The exclusion of these species from the legislation implies that the national legislation is not effective in their protection. It will also be difficult for other countries to act against the trade of these species as they are currently not protected or CITES-listed, just like the case of black-winged myna (*Acridotheres melanopterus*) in Indonesia [68]. The current South African legislation needs to be updated to include the trade of endemic, threatened, Critically Endangered, and Endangered species.

## 5. Limitations

We intentionally chose to focus on the trade of species not listed on CITES for this study. We concentrated on non-CITES species because they are less studied, and there is currently insufficient data within the South African context. By excluding CITES-listed taxa, we acknowledge that many species are indeed traded from South Africa to other countries, including through illegal trade, as indicated by the CITES dataset, which includes information on confiscations and wild-sourced species. Our dataset, however, did not include information on confiscations or trade sources since this information was not disclosed in available online resources and literature reviews. The non-CITES dataset lacks critical information such as seizures, confiscations, and the origin of the species (wild or captive-bred), creating significant gaps in our knowledge. For data collection, we relied on the LEMIS database, the literature review, and the online survey. The latter has constraints, as many listings use generic names without specifying species, and some platforms may not fully represent the trade, especially for non-CITES species. Identification of species based on online listings can be challenging due to generic names or lack of scientific nomenclature, potentially leading to misidentification or underestimation of species in the trade. Despite our thorough search strategy, we could have missed some important studies due to limited access to certain publications or the specific search terms used. This could have resulted in an incomplete picture of the species involved in the wildlife trade. The geographical scope is also one of the limitations; certain regions, particularly those with limited internet usage or alternative search engines, may not have been fully represented. For instance, major wildlife trading countries such as China predominantly use search engines other than Google, which could have affected the completeness of our data. Our focus on mammals, reptiles, birds, and amphibians excluded other groups, such as fish and insects, which may have omitted important trade aspects involving these taxa while maintaining a focused scope. The majority of online shops also focused on reptiles and amphibians, potentially skewing the representation of other taxa in our study. This may have resulted in an underrepresentation of species such as of mammals and birds in our dataset.

## 6. Conclusions

The existing data on non-CITES-listed species heavily rely on the commitment and transparency of importing countries to collect and disclose information publicly. Even though this is the case, our study was able to shed light on the trade of non-CITES-listed amphibians, birds, mammals, and reptiles. There is a huge demand for birds and mammals as wildlife products rather than as pets. However, this is not the case for amphibians and reptiles, which are more in demand as pets than as wildlife products. South Africa should include some of its amphibian species on the CITES database as none are currently listed on the international database. According to the NEM: BA regulation, indigenous species should not be traded and should be protected. We recommend that endemic and threatened species be protected and that their trade should be monitored, given that they are often potentially threatened by having a very limited range. Importing countries should ensure that South African trade regulations are being followed when harvesting species. In addition, the insufficient data on the trade of non-CITES-listed species poses a challenge to conservation initiatives, as the true extent of trade remains undisclosed. This is because the import data for non-CITES species depends on the importing countries. The USA is doing a great job in managing data for non-CITES species. This is evident from the comprehensive import data provided by the LEMIS dataset. The effectiveness of this database highlights the importance of similar systems globally. South Africa and other countries should consider developing a database that includes the trade of non-CITES species. This will be useful in tracking the trade volume of non-CITES species being traded both domestically and internationally. Accurate data on species trade can also inform conservation efforts and ensure sustainable use of wildlife resources. In addition, governments can use these data to create better policies and regulations to manage and protect biodiversity. The public also needs to be educated about the importance of conserving species with declining populations. A considerable responsibility also needs to be assigned to major consumers of non-CITES South African species. Major importing countries such as the USA, the Czech Republic, and the UK play an important role in driving the demand for South African species, particularly for pet trade purposes. As major consumers, these countries have responsibilities to ensure a sustainable trade, including implementing regulatory measures to prevent illegal trafficking. Given that CITES only enforces minimum measures on signatories, the South African government could tighten control over its wildlife by implementing a policy requiring permits for the movement of all wild animals within and from its borders, regardless of their CITES listing.

## Data Availability

All data used for this study are included in the Supplementary Materials.

## Supplementary Materials

The following supporting information can be downloaded at: https://www.mdpi.com/article/10.3390/ani14192782/s1, Figure S1: The number of CITES-listed and non-CITES-listed South African native taxa traded for pet trade and wildlife product purposes, both domestically and in international trade; Table S1: Inclusion criteria for species traded as pets and wildlife products; Table S2: Websites used in this study to identify South African species sold as pets; Table S3: Scientific papers used to identify South African species traded as pets and wildlife products; Table S4: South African species traded as pets, and their IUCN and TOPS status. The database from which the species were recorded included species highlighted in yellow, indicating endemic species in the country, and in green, indicating protected species in South Africa; Table S5: South African species traded as wildlife products, and their IUCN and TOPS status. The database from which the species were recorded included species highlighted in yellow, indicating endemic species in the country, and in green, indicating protected species in South Africa.
