# Supplementary material for: A Snapshot of the Global Trade of South African Native Vertebrate Species Not Listed on CITES
Source: Animals (Basel). 2024 Sep 26;14(19):2782. doi: 10.3390/ani14192782 (PMC11475766; doi:10.3390/ani14192782)
Supplement: Supplementary file 1 [file animals-14-02782-s001.zip › Shivambu et al. 2024_ Supplementary_Fig S1_TableS2 and S3.pdf]

Supplementary materials

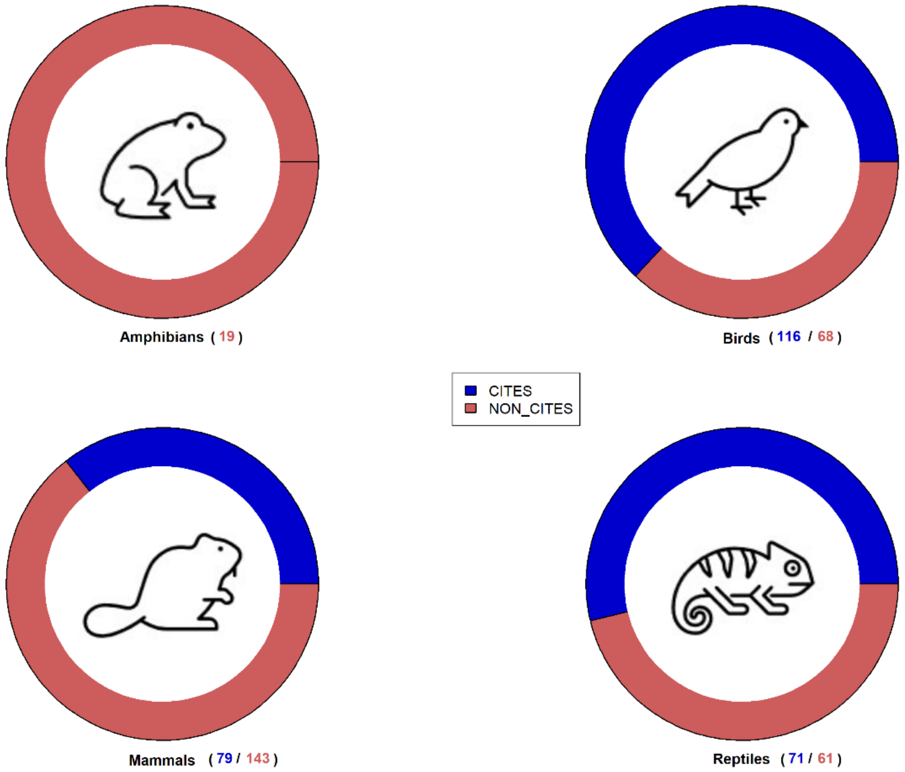

Supplementary Fig. S1. The number of CITES-listed and non-CITES-listed South African native taxa traded for pet trade and wildlife products purposes, both domestically and in international trade.

Supplementary Table S2. Websites used to identify South African species sold as pets in this study

**Country**	**Website**
Czech Republic	http://www.animalfarm.cz/
South Africa	https://wildlife4sale.co.za/
UK	https://www.exotic-pets.co.uk/
UK	https://crittery.co.uk/
UK	https://www.preloved.co.uk/
UK	https://potenzaexotics.com/
UK	https://fantasticfrogs.co.uk/
USA	https://mojavereptiles.com/
USA	https://jamjamexotic.com/

USA	https://americanreptiles.com/
USA	https://www.lllreptile.com/
USA	https://joshsfrogs.com/
USA	https://www.backwaterreptiles.com/
USA	https://www.exoticanimalsforsale.net/
USA	https://imperialreptiles.com/
USA	https://www.petzlover.com/us/
USA	https://theturtlesource.com/
USA	https://www.faunaclassifieds.com/forums/index.php?
USA	https://snakesatsunset.com/
Germany	https://www.terraristik.com/

Supplementary Table S3. Scientific papers used to identify South African species traded as pets and wildlife products.

**Pet trade**		
**Country**	**Paper title**	**Publication year**
Australia	Detecting and preventing new incursions of exotic animals in Australia	2011
Australia	Patterns of transport and introduction of exotic amphibians in Australia	2014
Czech Republic	Potential Invasion Risk of Pet Traded Lizards, Snakes, Crocodiles, and Tuatara in the EU on the Basis of a Risk Assessment Model (RAM) and Aquatic Species Invasiveness Screening Kit (AS-ISK)	2019
Germany	Strategies to reduce demand for reptiles kept as pets, Amphibians and small mammals Species Conservation Relevance of the Pet Trade	2017
Germany	The Rush for the Rare: Reptiles and Amphibians in the European Pet Trade	2020
Germany	Endoparasites infecting exotic captive amphibian pet and zoo animals (Anura, Caudata) in Germany	2020
Italy	Survey of *Hymenolepis* spp. in pet rodents in Italy	2015
Japan	*Batrachochytrium dendrobatidis* prevalence and haplotypes in domestic and imported pet amphibians in Japan	2014
Japan	Exotic animal cafés in Japan: A new fashion with potential implications for biodiversity, global health, and animal welfare	2023
Japan	Exotic animal cafes are increasingly home to threatened biodiversity	2021

Pet trade		
**Country**	**Paper title**	**Publication year**
Philippines	Checklist of exotic species in the Philippine pet trade, II. Reptiles	2015
Philippines	Checklist of exotic species in the Philippine pet trade, i. amphibians	2014
Republic of Korea	Present Status of Non-Native Amphibians and Reptiles Traded in Korean Online Pet Shop	2020
South Africa	Online and pet stores as sources of trade for reptiles in South Africa	2022
South Africa	An assessment of avian species sold in the South African pet trade	2022
South Africa	Non-native small mammal species in the South African pet trade	2021
Taiwan	Exotic Amphibians in the Pet Shops of Taiwan	2006
UK	Emerging Infectious Disease and the Trade in Amphibians; pet retailers survey	2014
UK	Risk assessment model for the import and keeping of exotic reptiles and amphibians	2005
USA	Amphibian and reptile trade in Texas: current status and trends	2010
USA	Where do African clawed frogs come from? An analysis of trade in live *Xenopus laevis* imported into the USA	2017
USA	Risk assessment model for the import and keeping of exotic reptiles and amphibians	2005
South Africa	Alien invaders and reptile traders: what drives the live animal trade in South Africa?	2010
**Wildlife trade**		
**Country**	**Paper title**	
South Africa	Animals traded for traditional medicine at the Faraday market in South Africa: species diversity and conservation implications	2013
South Africa	Risks to Birds Traded for African Traditional Medicine: A Quantitative Assessment	2014
South Africa	Wildlife Trade for Belief-Based Use: Insights From Traditional Healers in South Africa	2022
South Africa	Conservation implications of the use of vertebrates by Xhosa traditional healers in South Africa	1998
South Africa	Traditional medicinal animal use by Xhosa and Sotho communities in the Western Cape Province, South Africa	2019
